# Preparation, Characterization, and Enhanced Thermal and Mechanical Properties of Epoxy-Titania Composites

**DOI:** 10.1155/2014/515739

**Published:** 2014-01-20

**Authors:** Zakya Rubab, Adeel Afzal, Humaira M. Siddiqi, Shaukat Saeed

**Affiliations:** ^1^Department of Chemistry, Quaid-i-Azam University, Islamabad 45320, Pakistan; ^2^Dipartimento di Chimica, Università degli Studi di Bari, Via Orabona 4, 70126 Bari, Italy; ^3^Affiliated Colleges at Hafr Al-Batin, King Fahd University of Petroleum and Minerals, P.O. Box 1803, Hafr Al-Batin 31991, Saudi Arabia; ^4^Department of Metallurgy and Materials Engineering, Pakistan Institute of Engineering and Applied Sciences, Islamabad 45650, Pakistan

## Abstract

This paper presents the synthesis and thermal and mechanical properties of epoxy-titania composites. First, submicron titania particles are prepared via surfactant-free sol-gel method using TiCl_4_ as precursor. These particles are subsequently used as inorganic fillers (or reinforcement) for thermally cured epoxy polymers. Epoxy-titania composites are prepared via mechanical mixing of titania particles with liquid epoxy resin and subsequently curing the mixture with an aliphatic diamine. The amount of titania particles integrated into epoxy matrix is varied between 2.5 and 10.0 wt.% to investigate the effect of sub-micron titania particles on thermal and mechanical properties of epoxy-titania composites. These composites are characterized by X-ray photoelectron (XPS) spectroscopy, scanning electron microscopy (SEM), differential scanning calorimetry (DSC), thermogravimetric (TG), and mechanical analyses. It is found that sub-micron titania particles significantly enhance the glass transition temperature (>6.7%), thermal oxidative stability (>12.0%), tensile strength (>21.8%), and Young's modulus (>16.8%) of epoxy polymers. Epoxy-titania composites with 5.0 wt.% sub-micron titania particles perform best at elevated temperatures as well as under high stress.

## 1. Introduction

In past few years, major research efforts have been focused on the development of high performance composite materials based on organic polymers and inorganic particles with key emphasis on developing methods to better integrate (or disperse) inorganic fillers into organic polymers. Inorganic filler particles, for example, silica [1], zirconia [2], and titania [3], are widely used to enhance the mechanical [4–6], electrical [7], optical [8], and thermal [9, 10] properties of epoxy polymers. These properties of the organic-inorganic composites are fundamentally determined by the distribution and the size of inorganic filler particles and their interaction with the organic polymer [5, 11]. Albeit several experimental approaches and processing conditions have already been reported in the literature to prepare thermally and/or mechanically superior composite materials [12–14], the development of a universally simple method to prepare inorganic filler particles and to improve their distribution in organic polymeric matrix such as epoxy resins remains a challenge. Herein, we report a simple and straightforward route to prepare submicron titania particles without the use of surfactants or surface modifiers. These particles are later used as inorganic fillers for epoxy resins to enhance their thermal and mechanical performance.

To date, the performance of titania fillers in different polymeric matrices especially in epoxy resins has not been fully explored. There are only a few reports describing the behavior of submicron titania epoxy composites. For instance, commercially obtained submicron titania particles (size: ~190 nm) have been incorporated in epoxy resin to study mechanical properties of the resulting composite materials [15]. It was concluded that epoxy-titania composites with 4 wt.% submicron titania particles exhibit the highest toughness and impact resistance. A more detailed literature survey indicates that titania is not frequently used as a filler for polymeric matrices [3, 16–18]. We are particularly interested in exploring titania as a filler for epoxy polymers because the mechanical properties of titania and titania doped (filled) materials are far better than silica—one of the most frequently used fillers—and titania is thermally as good as silica [19–21].

Our aim is to enhance thermal and mechanical properties of epoxy polymers using titania filler and to understand the behavior of these composites at elevated temperature and under high stress. Thus, the paper presents synthesis of epoxy-titania composites via direct blending (mechanical mixing) of the as-prepared, submicron titania particles with epoxy resin monomers and subsequently curing the mixtures with a flexible diamine hardener. These composites are synthesized in the form of thin films, that is, 0.8–1.0 mm thick, and contain different ratios (wt.%) of titania particles. Thermal and mechanical properties of epoxy-titania composites are determined and reported with reference to neat epoxy polymer.

## 2. Experimental

### 2.1. Materials

Diglycidy lether of bisphenol A (DGEBA, molar mass: 348.5 g mol^−1^, Dow Chemicals), poly(oxypropylene) diamine (POPDA, molar mass: 398 g mol^−1^, Huntsman Co.), and titanium tetrachloride (TiCl_4_, Merck) were used as precursors for preparing epoxy polymer and titania particles, respectively. All chemicals were used as received without further purification.

### 2.2. Synthesis

Two types of polymer thin films were synthesized: (a) neat epoxy polymer as a reference material for performance comparison of (b) epoxy-titania composites with different ratios (wt.%) of titania particles. The detailed procedures to prepare neat epoxy polymer, titania particles, and epoxy-titania composites are given below.

#### 2.2.1. Synthesis of Neat Epoxy Polymer

Neat epoxy polymer thin films were prepared by mixing equimolar quantities of DGEBA and POPDA for 1 hour at room temperature. The mixture was subsequently poured into PTFE molds especially designed to prepare polymer films with less than 1 mm thickness. The material was degassed to remove air bubbles and then cured in an oven at 100°C for 5 hours.

#### 2.2.2. Synthesis of Titania Particles

To a 100 mL two-neck round bottom flask fitted with a septum, a condenser, and a magnetic bar, 40 mL of ethanol and 5 mL of water were added. TiCl_4_ (0.6 mL, 0.05 mol) was injected through the septum, while the mixture was being stirred continuously. The reaction mixture was stirred at 60°C for half an hour. Liquid ammonia (5 mL, 0.1 M) was subsequently injected dropwise through the septum to attain a pH of 8. The mixture was aged for 6 hours at room temperature with persistent magnetic stirring. The product (white precipitates) was washed first with water (5 mL) and then with acetone (5 mL). Solid particles were recovered by centrifugation at 6000 rpm. Finally, the product was dried under vacuum at 120°C to yield 0.2 g of titania particles. These particles were subsequently used to prepare epoxy-titania composites without further heat treatment (or calcination). Thermal treatment of titania particles was avoided intentionally to leave a sufficient number of hydroxyl (–OH) groups on the surface of particles for potential weak interactions with the organic matrix.

#### 2.2.3. Synthesis of Epoxy-Titania Composites

To a 25 mL Erlenmeyer flask, DGEBA (0.02 mol) and the calculated amounts of titania particles were added. The mixture was stirred mechanically for 1 hour to effectively disperse titania particles. Finally, stoichiometric amount of POPDA (0.01 mol) was added to the mixture, and stirring was continued for 1 hour. The mixture was casted in PTFE molds and subsequently cured at 100°C for 5 hours. The amount of titania particles integrated into the matrix polymer was varied between 0.3 and 1.1 g to prepare epoxy-titania composites with 2.5, 5.0, 7.5, and 10.0 wt.% titania loadings. The epoxy-titania composite samples were named as ET-2.5, ET-5, ET-7.5, and ET-10 composites, respectively, conferring to the amount of titania particles (in wt.%) dispersed into epoxy polymers.

### 2.3. Methods

Neat epoxy polymer and epoxy-titania composites are characterized with the help of X-Ray Photoelectron Thermo VG Theta Probe spectrometer, equipped with a microspot monochromatized Al K*α* source. The XPS survey spectra were acquired in fixed analyzer transmission mode with pass energy of 150 eV.

Microscopic characterization of thin films was carried out on JEOL JSM-5910 scanning electron microscope (SEM) equipped with tungsten filament electron emitter at accelerating voltage of 30 kV.

Differential scanning calorimetry (DSC) of composite samples was performed on PerkinElmer DSC 7 instrument by heating samples in a sealed Al pan under inert (N_2_) atmosphere. The instrument was ramped at 10°C min^−1^ from −80°C to 300°C.

Thermogravimetric (TG) analyses were performed on PerkinElmer TGA 7 instrument. The samples were heated from room temperature to 700°C at the rate of 20°C min^−1^ in air, and weight loss was monitored. The data obtained from TG curves were filed into a curve fitting software—*the Battery Program* [9]—to calculate the energy of activation (*E*_*a*_) of pyrolysis for neat polymer and epoxy-titania composites.

Stress-strain curves of composite films were obtained on Testometric Universal Testing Machine M350/500. The samples for mechanical analysis were vacuum dried at 60°C for 2 hours before tensile measurements to remove adsorbed moisture, if any. The tensile tests were performed at 25°C with a constant crosshead speed of 1 mm min^−1^ [22]. All measurements, that is, DSC, TG, and mechanical analyses, were performed in triplicate at least to report precise values.

## 3. Results and Discussion

### 3.1. Characterization

**Figure 1:**
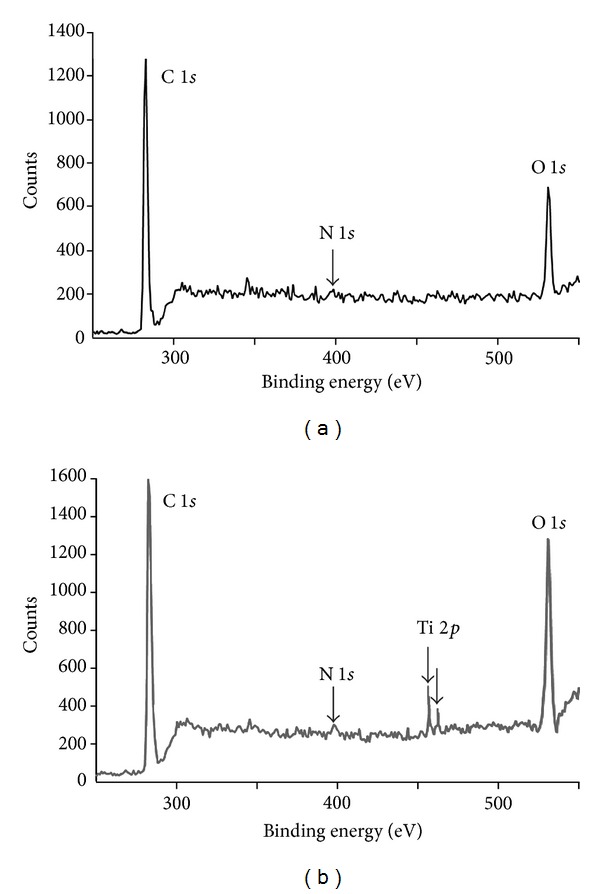
X-ray photoelectron spectra of neat epoxy polymer and ET-10 composite films.

X-ray photoelectron spectroscopy is a powerful tool to characterize neat and titania filled epoxy polymers and to determine their surface elemental composition.  Figure 1 shows the XPS survey scans of neat polymer and epoxy-titania composites with 10 wt.% titania. Carbon (C 1*s*) and oxygen (O 1*s*) peaks are observed around 284.8 and 531.5 eV [23], respectively (in Figure 1(a)), indicating the presence of these elements. Nitrogen (N 1*s*) peak around 399 eV is not clear due to very low percentage of nitrogen atoms in the polymer. In contrast, the XPS survey scan of ET-10 composite demonstrates the presence of titanium (Ti 2*p*) peak—a doublet around 459.2 eV [24]—in addition to characteristic C 1*s* and O 1*s* peaks (in Figure 1(b)), which confirms the presence of titania particles in epoxy matrix. The inclusion of titania is also evident through enhanced O 1*s* peak intensity in Figure 1(b) that means a higher number of surface oxygen atoms are detected in case of ET-10 composite as compared to neat polymer.

XPS spectra may also reveal information about crystalline state of titania particles. According to NIST XPS database [23] and published reports [24], crystalline titania peak is observed at slightly lower binding energy (458.5 eV) as compared to amorphous titania (at 459.2 eV). Thus, XPS spectrum of ET-10 composite indicates the presence of amorphous titania particles. This is an expected result since as-prepared titania particles are used as fillers, that is, without prior heat treatment or calcination, which is an essential step for crystallization of metal oxides prepared via sol-gel process [25].

**Figure 2:**
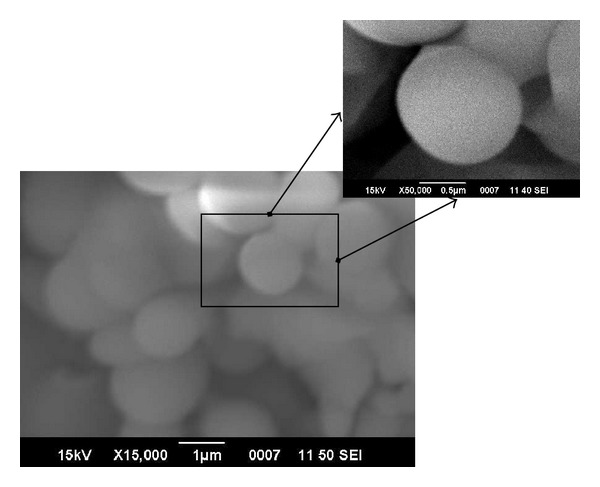
SEM images of submicron titania particles: the size of particles is in the range of 600–800 nm.

SEM images of titania particles as well as epoxy-titania composites were obtained to study the morphology, size, and shape of titania particles and their distribution in the polymeric matrix. SEM images of titania particles are shown in Figure 2. The presence of relatively large, spherical titania particles is confirmed by SEM. The size of these particles is in the submicron regime, varying between 600 and 800 nm (0.6–0.8 *μ*m). SEM image of titania particles demonstrates that these particles have good dispersity, and they are not severely agglomerated although no surfactant or surface modifier was used.

**Figure 3:**
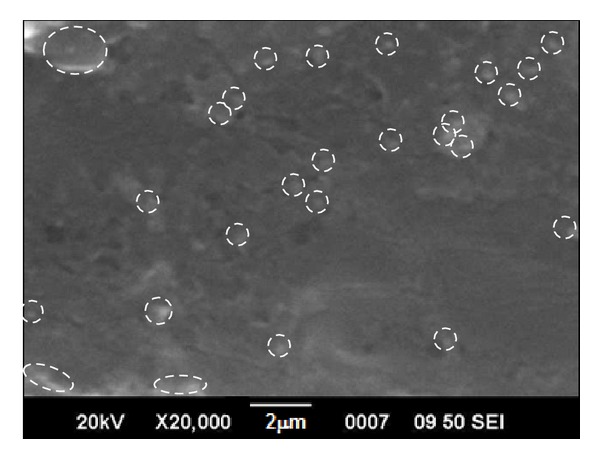
SEM image of epoxy-titania composite films with 5 wt.% titania particles (ET-5).

Figure 3 shows the SEM image of epoxy-titania composite surface containing 5 wt.% submicron titania particles. The micrograph exhibits a fairly rough surface morphology and spherical titania particles can be identified on the surface of composite film. It is evident that these titania particles are homogenously distributed in the epoxy matrix. Although titania particles, when mixed or blended with viscous epoxy resins, tend to agglomerate (or form clusters), the overall size distribution of titania particles is observed to be narrow and the overall dispersion of particles within epoxy polymer is quite uniform. Furthermore, titania particles are not seen as protrusions or sovereign particles lying of the surface, but they seem to be well embedded into the matrix showing good particle-matrix adhesion, which may be attributed to weak physical interactions such as H-bonding between the surface –OH groups of titania particles and the –OH groups present on the polymer backbone. Since as-prepared amorphous titania particles are dispersed in the epoxy resin, –OH groups present on the surface of these particles lead to better interphase interactions.

### 3.2. Curing Behavior and Glass Transition Temperature

**Figure 4:**
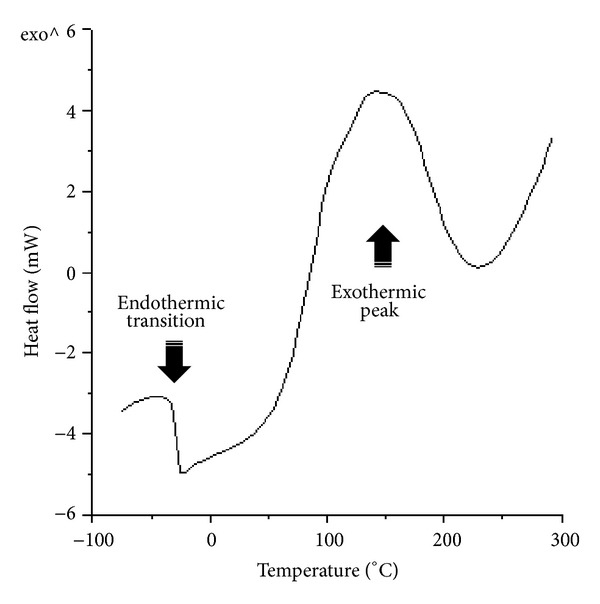
DSC scan of uncured epoxy-titania composition (ET-10): endothermic and exothermic transitions are clearly visible.

DSC was used to study curing behavior and glass transition temperature (*T*_*g*_) of neat epoxy polymer as well as epoxy-titania composites.  Figure 4 shows DSC scan of an uncured epoxy-titania composite mixture containing 10 wt.% titania particles (ET-10). Two transitions are clearly observed in Figure 4: the first one is a small endothermic shift corresponding to the glass transition of uncured sample, while second one shows a huge exotherm in the range of 50–250°C that is attributed to the ring opening reaction between amino and epoxy monomers [14]. All epoxy-amine mixtures are therefore cured at 100°C for 5 hours to achieve complete crosslinking of epoxide groups with the amine monomers.

**Figure 5:**
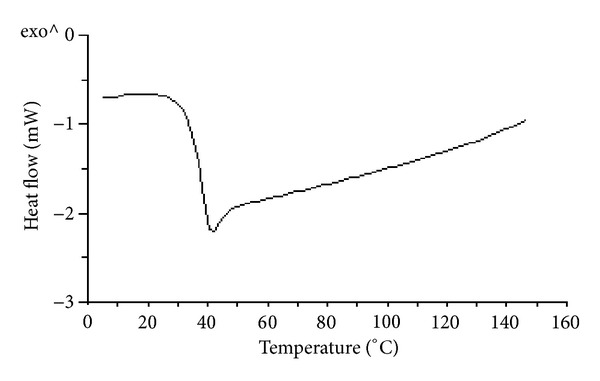
Typical DSC curve of ET-10 composite showing glass transition region.

Figure 5 shows typical DSC scan of the same epoxy-titania (ET-10) composite after curing. In contrast to Figure 4, DSC scan presented in Figure 5 does not show an exothermic transition above 100°C, which confirms that the epoxide ring opening reaction (crosslinking) with the diamine is completed during the curing cycle. In addition, the glass transition region is shifted from below zero (in Figure 4) to above 40°C (in Figure 5) that also indicates complete crosslinking of the respective monomers. Initially, the mixture of monomers shows *T*_*g*_ below 0°C since monomers (or oligomers, if any) can freely move at this stage. However, once all monomers have reacted to form a three-dimensional polymer network, the movement of polymeric chains is considerably hindered leading to an increase in *T*_*g*_ after curing.

DSC scans of epoxy-titania composites with different loadings of titania particles also reveal a variable shift in *T*_*g*_ of epoxy polymers. At first, in comparison with neat epoxy polymer, *T*_*g*_ of epoxy-titania composites increases slightly at low loadings of titania particles, for example, 2.5 and 5.0 wt.%, which suggests that the polymeric matrix and the titania filler interact favorably at low filler concentration leading to a structure with improved *T*_*g*_. However, as given in Table 1, a sharp decrease in *T*_*g*_ of epoxy-titania composites is observed at higher loadings of titania particles, that is, 7.5 and 10.0 wt.%. This may be attributed to the increased distance between polymeric chains and lower crosslink density. At higher concentration of submicron titania particles, bigger particles tend to force polymeric chains to be apart and reduce the cohesive energy density [26]. This phenomenon also results in a rapid increase in the free volume of epoxy polymers, as reported by different authors [27]. It can be said that *T*_*g*_ of epoxy-titania composites decreases at higher loadings of titania particles.

### 3.3. Thermal and Mechanical Properties

**Figure 6:**
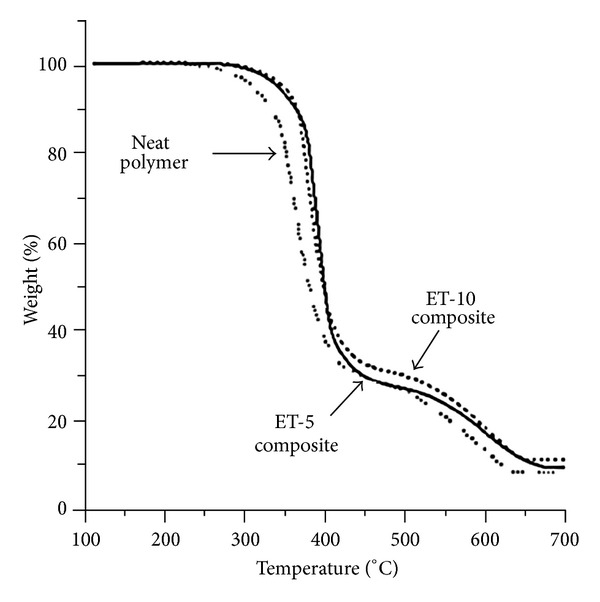
Typical TG scans of neat epoxy polymer and epoxy-titania composites with different concentrations of submicron titania particles.

TG analyses of epoxy-titania composites are performed to investigate thermal degradation and high temperature stability of these composites with reference to neat epoxy polymer. TG analyses of neat polymer and composite samples demonstrate a substantial increase in thermal decomposition temperatures as compared to neat epoxy polymer.  Figure 6 shows typical TG curves (thermograms) of neat epoxy polymer and epoxy-titania composites with different loadings of submicron titania particles. The data collected from at least three TG measurements of each sample are reported in Table 1.

Thermal degradation temperatures such as initial decomposition temperature (IDT) and *T*_*d*10_ and *T*_*d*50_ corresponding, respectively, to 10% and 50% weight loss are significantly increased at higher loadings of titania particles. It is evident that epoxy-titania composite with 7.5 wt.% titania (ET-7.5) shows the highest degradation temperatures. At 10 wt.% titania loading, *T*_*d*10_ and *T*_*d*50_ are slightly decreased, which may be attributed to the most likely microscopic phase separation between epoxy polymer and titania particles due to reduced interphase interactions. The microscopic phase separation between organic and inorganic phases cannot be avoided, though it may be delayed to a higher loading of inorganic filler by improving interphase interactions [14]. In our experiments, the interface is based on weak interactions, for example, between –OH groups, which may not be favorable at high concentrations of titania.

Nonetheless, the temperature at which the rate of weight loss is maximum, referred to as *T*_ max _, is increased as a function of titania content, and epoxy-titania composite with 10 wt.% titania (ET-10) shows the highest *T*_ max_ values as compared to neat epoxy polymer and other epoxy-titania composites. The energy of activation (*E*_*a*_) for pyrolysis, as computed through *Battery Program* [9], also increases with the increase in titania content, which means that the rate of degradation is slower at higher concentration of titania particles [28]. Hence, it can be concluded that the addition of submicron titania particles into epoxy polymers significantly enhances thermal stability of resulting epoxy-titania composites.

The average char yields or residues at 700°C, as measured from the respective TG traces, also increase from 8% for neat polymer to 10.5% and 12.5% for ET-5 and ET-10 composites, respectively. However, this increment in char yield is relatively smaller compared to the amount of titania incorporated into the polymer. This may be due to the presence of amorphous titania particles, which loose surface –OH groups at elevated temperature leading to a reduction in the wt.% of titania particles at 700°C. In addition, as discussed earlier, the inclusion of submicron titania particles in the polymer forces the polymeric chains to be apart thus reducing the cohesive energy density and degree of crosslinking, which may also result in a higher rate of degradation of polymeric chains thus leaving behind lower amount of organic residue compared to neat polymer.

**Figure 7:**
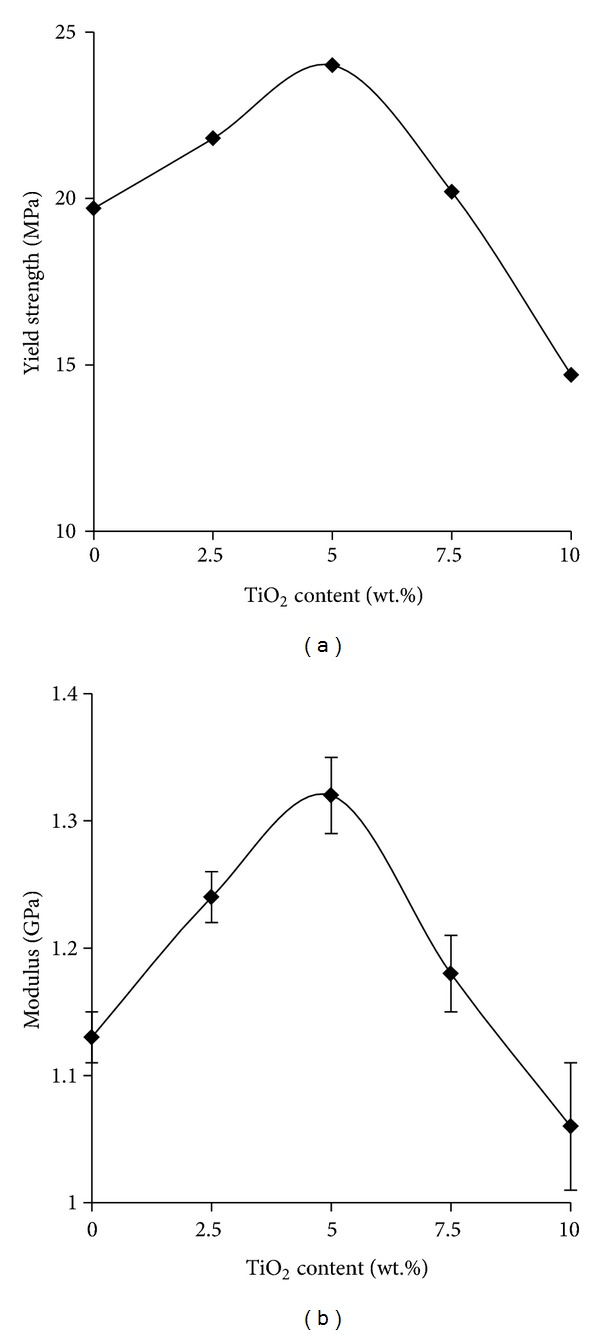
Mechanical properties of epoxy-titania composites as a function of the concentration of titania particles.

Mechanical properties of neat epoxy polymer and epoxy titania composites were determined to investigate the effect of submicron titania particles on strength and stiffness of epoxy polymer.  Figure 7 shows the mechanical properties of different epoxy polymers as a function of titania content determined from stress-strain curves. It is found that epoxy-titania composites possess superior mechanical properties when compared to neat epoxy polymer. It is obvious that both yield (tensile) strength and Young's modulus are significantly enhanced at 2.5 and 5.0 wt.% loadings of titania particles.

The performance of epoxy-titania composites is decreased at relatively higher concentrations of titania particles, that is, at 7.5 and 10.0 wt.% titania. Considering the microstructure of epoxy-titania composites, this behavior can be explained by understanding the initiation and propagation of microcracks during mechanical analysis. According to pertinent literature [4, 29], once these microcracks encounter filler particles, they are dispersed depending upon the interface strength and size of particles. In case of submicron titania particles, this may lead to the formation of microvoids as soon as stress is increased [15]. Thus, at higher concentration of titania in epoxy polymer with only weak interfacial interactions, the specimen breaks at lower stress and exhibits relatively lower yield strength as compared to similar epoxy-titania composites with lower concentrations of titania particles. These results are in good agreement with the reported literature [11, 15, 30].

## 4. Conclusions

In conclusion, the paper presented synthesis of submicron titania particles with sizes in the range of 0.7–0.8 *μ*m via surfactant-free sol-gel method. The inorganic particles were subsequently dispersed into epoxy polymer matrix to prepare epoxy-titania composites with different concentrations of submicron titania particles, that is, 2.5–10.0 wt.%. The resulting epoxy-titania composites were characterized by XPS and SEM. DSC and TG results revealed significant enhancements in the glass transition (*T*_*g*_) and thermal degradation temperatures of epoxy-titania composites when compared to neat epoxy polymer. Mechanical analysis of composite films also exhibited a maximum of 17% increase in Young's modulus of epoxy-titania composites with 5 wt.% titania particles (ET-5) with reference to neat epoxy polymer. Albeit the results were not promising at relatively higher loadings of submicron titania particles, thermal and mechanical properties of epoxy-titania composites were effectively improved at 2.5–7.5 wt.% titania loadings. It was observed that ET-5 composite had superior properties and generally performed best.

## Figures and Tables

**Table 1:** Thermal properties of neat epoxy polymer and epoxy-titania composites with different concentrations of submicron titania particles.

Sample	*T*_*g*_ (°C)	IDT (°C)	*T*_*d*10_ (°C)	*T*_*d*50_ (°C)	*T*_max_ (°C)	*E*_*a*_ (kJ·mol^−1^)
Neat polymer	45	291	326	376	362, 566	108
ET-2.5 composite	48	299	351	385	372, 577	135
ET-5 composite	46	307	359	391	379, 585	147
ET-7.5 composite	44	313	365	395	383, 591	155
ET-10 composite	41	315	361	393	388, 599	159
